# Development and External Validation of a Simple-To-Use Dynamic Nomogram for Predicting Breast Malignancy Based on Ultrasound Morphometric Features: A Retrospective Multicenter Study

**DOI:** 10.3389/fonc.2022.868164

**Published:** 2022-04-07

**Authors:** Qingling Zhang, Qinglu Zhang, Taixia Liu, Tingting Bao, Qingqing Li, You Yang

**Affiliations:** ^1^Depatment of Ultrasonography, The First Affiliated Hospital of Wannan Medical College, Wuhu, China; ^2^Department of Ultrasonography, Shandong Provincial Third Hospital Affiliated to Cheeloo College of Medicine, Shandong University, Jinan, China; ^3^Department of Ultrasonography, Linyi People’s Hospital, Linyi, China

**Keywords:** breast cancer, models, quantitative imaging, nomogram, morphometrics, ultrasound

## Abstract

**Background:**

With advances in high-throughput computational mining techniques, various quantitative predictive models that are based on ultrasound have been developed. However, the lack of reproducibility and interpretability have hampered clinical use. In this study, we aimed at developing and validating an interpretable and simple-to-use US nomogram that is based on quantitative morphometric features for the prediction of breast malignancy.

**Methods:**

Successive 917 patients with histologically confirmed breast lesions were included in this retrospective multicentric study and assigned to one training cohort and two external validation cohorts. Morphometric features were extracted from grayscale US images. After feature selection and validation of regression assumptions, a dynamic nomogram with a web-based calculator was developed. The performance of the nomogram was assessed with respect to calibration, discrimination, and clinical usefulness.

**Results:**

Through feature selection, three morphometric features were identified as being the most optimal for predicting malignancy, and all regression assumptions of the prediction model were met. Combining all these predictors, the nomogram demonstrated a good discriminative performance in the training cohort and in the two external validation cohorts with AUCs of 0.885, 0.907, and 0.927, respectively. In addition, calibration and decision curves analyses showed good calibration and clinical usefulness.

**Conclusions:**

By incorporating US morphometric features, we constructed an interpretable and easy-to-use dynamic nomogram for quantifying the probability of breast malignancy. The developed nomogram has good generalization abilities, which may fit into clinical practice and serve as a potential tool to guide personalized treatment. Our findings show that quantitative morphometric features from different ultrasound machines and systems can be used as imaging surrogate biomarkers for the development of robust and reproducible quantitative ultrasound dynamic models in breast cancer research.

## Introduction

Globally, breast cancer is the leading cause of cancer-associated death in women (1). Effective screening approaches have the ability to reduce cancer-related mortality rates (2, 3). Due to its safety and wide availability, US examination is recommended as a supplemental screening tool for women of all ages (4). In asymptomatic women, the ability of US to detect breast cancer is comparable to that of mammography (5–7). Over the years, a structured reporting and classification system has been widely adopted for qualitatively describing breast US findings in routine clinical practice (e.g., ACR BI-RADS) (8). However, image interpretation for the traditional structured classification is generally subjective and is possibly affected by radiologists’ experience (9–11). Moreover, predictions of malignancies by the classification system are not always precise, and there are significant differences between hospitals. As reported in the literature, BI-RADS category 4 lesions have a broad range of malignancy rates (3-94%) (12). Thus, the US capacity for detecting breast malignancy still needs to be upgraded considerably.

In the precision medicine context, quantitated methods provide the unique potential for making breast cancer screening more rapid and accurate using artificial intelligence and machine learning algorithms (13). Many studies are evaluating the applicability of US prediction models that are based on quantitated methods (e.g., radiomics) (14–17). These models have been developed to mine high-throughput quantitative image features fusing image pixels and morphology through machine learning methods to improve cancer diagnosis and prognosis (18). However, to varying degrees, reproducibility of quantification features derived from image pixels is sensitive to image preprocessing (19), particularly for US technology, which has the distinct inherent characteristic of operator- and device-dependent, not to mention that such pixel-based features often lack interpretability (20). This may lead to limitations in usability for real end-users, impeding their large-scale clinical applications.

Morphometrics, which are associated with tumor histological findings (21), refers to the quantified assessment of shape variations of organisms and their covariations with other variables. Unlike image pixel-based features, morphometric features characterize the shape and contour of lesions and are nearly independent of the different system settings and US machines (22). We hypothesized that a set of quantified morphological features are related to malignant breast lesions and may, therefore, act as independent predictive markers, without the involvement of pixels-based features. We tested this hypothesis and further build an interpretable and simple-to-use US nomogram for predicting breast malignancy.

## Materials and Methods

### Study Population

In this multicenter retrospective study, patients were recruited from three tertiary medical centers; The First Affiliated Hospital of Wannan Medical College in Anhui Province (Center A), Shandong Provincial Third Hospital Affiliated to Cheeloo College of Medicine, Shandong University (Center B), and Linyi People’s Hospital in Shandong Province (Center C). The training cohort for nomogram development was obtained from among the patients at Center A between January 2020 and September 2021 while the external validation cohorts were derived from Centers B and C between January 2021 and September 2021.

All consecutive female patients with US findings of breast lesions who fulfilled the inclusion/exclusion criteria were enrolled. The inclusion criteria were: i. The definitive pathological diagnosis was available from the breast lesion, either by biopsy or surgery; ii. US examination performed before biopsy or surgery; and iii. Breast lesions classified as BI-RADS US category 4 or 5 according to the second edition of the ACR BI-RADS US atlas. The exclusion criteria were: i. Indeterminate pathological results (difficult to distinguish between “benign” and “malignant”), ii. Incomplete clinical information, iii. Patients administered with radiotherapy or chemotherapy before US examination, and iv. Patients whose longest diameter of the lesion was beyond the display range of the US transducer. For patients with more than one lesion, only the lesion with confirmed pathological diagnosis was included for quantitative analysis.

The First Affiliated Hospital of Wannan Medical College Review Board, Shandong Provincial Third Hospital Review Board and Linyi People’s Hospital Review Board approved this retrospective study. Patient consent was waived due to the use of retrospective, de-identified information from the image database.

### Ultrasound Examination

Five different high-resolution US scanners equipped with a linear array transducer, including Esaote Mylab 90 (Genova, Italy) with a 4-13 MHz transducer, Siemens Acuson S3000 (IL, USA) with a 4-9 MHz transducer, Philips IU22 (PA, USA) with a 3-12 MHz transducer, Philips EPIQ5 (PA, USA) with a 5-12 MHz transducer, and Mindray Resona 7T (SZ, China) with 5-14 MHz transducer were used in this study.

All lesions were examined by 7 sonographers who had over 5 years of experience in breast US scanning. Parameters were adjusted to optimize image quality, then, the grey-scale image of the longest diameter section of target lesions was documented in the JPG format for further quantification analysis.

### Outcome Measures

The outcome was the definitive histopathologic diagnosis by biopsy or surgery. Pathological results were reported independently by the pathologist of the participating hospitals and grouped into malignant and benign lesions. Histological processing was performed in the accredited Department of Pathology and conducted using a standardized procedure to ensure reproducibility.

### Data Quality Control

Imaging and clinical data were collected by an independent investigator from respective hospitals. A radiologist with more than eight years of experience in breast US reviewed the results of data collecting and further confirmed the final datasets according to the inclusion/exclusion criteria. These data were anonymized and randomly attached with a number ID. Images of benign and malignant lesions from the training cohort were mixed and stored in a single folder for quantification analysis, so were those from the validation cohorts.

Morphometric analyses of images were independently performed by three sonographers who were not involved in data collection. Three identical lap-tops with 1920 × 1280 resolution were used, and each image was magnified by the delineate process so that the lesion occupied at least half of the display area. Lesions from the training cohort were measured by sonographer QL, while those from the validation cohorts were respectively measured by sonographers TB and YY. All the sonographers had 4 years of work experience in breast US. At the beginning of the study, they were uniformly trained on the use of the image quantification software. In addition, they were blinded to the clinical information and pathologic results as well as on the ratios of malignant to benign lesions.

### Morphometric Feature Extraction

Image morphometric analysis was performed using the ImageJ software (https://imagej.nih.gov/ij, version 1.52p, NIH, USA). First, grey-scale US images of all target lesions were exported from the machines and imported into the ImageJ software. For each lesion, only one image was extracted. Next, using the Set Scale function of the Analyze Tab menu in ImageJ, lesion sizes were calibrated according to depth bar on each US image to obtain the actual size value. Finally, the contour of each lesion was manually delineated as the region of interest (ROI).

**Figure 1:**
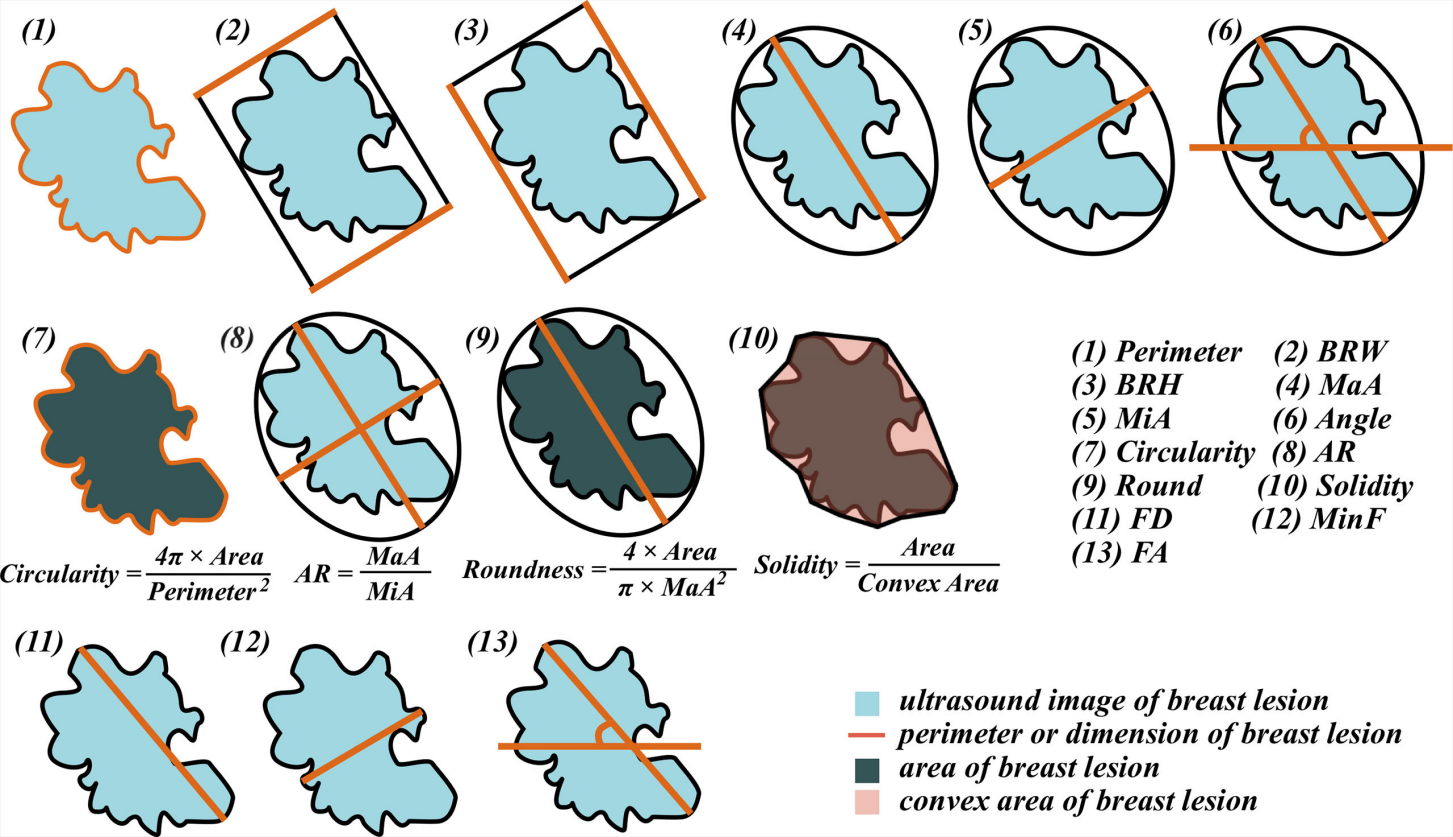
Illustrations of the morphometric features.

After delineating the ROI of lesions, thirteen morphometric features were automatically calculated and extracted: (1) Perimeter, the length of the outside boundary of the ROI; (2) Bounding Rectangle Width (BRW), the width of the smallest rectangle enclosing the ROI; (3) Bounding Rectangle Height (BRH), the height of the smallest rectangle enclosing the ROI; (4) Major Axis (MaA), the primary axis of the best fitting ellipse to the ROI; (5) Minor Axis (MiA), the secondary axis of the best fitting ellipse to the ROI; (6) Angle, the angle between the Major Axis and a line parallel to the x-axis of the US image, its range is 0 -180 degrees; (7) Circularity, a morphological feature that can mathematically indicate the degree of similarity to a perfect circle, taking into consideration the smoothness of the perimeter. This means that circularity is a measure of both lesion shape and roughness, the further away from a perfectly round and smooth circle, the lower the circularity value of the target lesion; (8) Axis Ratio (AR), the ratio of Major Axis and Minor Axis; (9) Roundness (Round), a value of 1.0 indicates a perfect circle. It is similar to circularity but is insensitive to irregular borders along the perimeter of the target lesion, also takes into consideration the major axis of the best fit ellipse; (10) Solidity, the ratio of contour area to its convex hull area, describes the extent to which a target lesion morphology is convex or concave. As lesion morphology becomes rough, the solidity value approaches zero. Conversely, very smooth, rounded lesions have solidity values that approach one; (11) Feret Diameter (FD), the longest distance between any two points along the ROI boundary, also known as a maximum caliper; (12) Min Feret (MinF), the minimum caliper diameter; (13) Feret Angle (FA), the angle between the Feret Diameter and a line parallel to the x-axis of the US image, its range is 0 -180 degrees. **Figure 1** shows illustrations of all the morphometric features.

### Assessment of Intra- and Inter-Rater Reliability

Based on the calculated sample size (23), 80 lesions from the training cohorts were randomly selected to assess intra- and inter-rater reliability. Using the same procedure, the original assessor QL and another assessor TB performed the second measurements, three weeks after the first one.

### Feature Selection

Feature selection was performed on the training cohort. A two-step feature selection procedure was used to generate optimal feature subsets. First, the features were ranked by the wrapper method *Boruta* algorithm (24). *Boruta* assesses if the importance of each individual feature is significantly higher than the importance of a random feature by iteratively fitting the Random Forest algorithm until all predictor features are classified as “confirmed,” “tentative,” or “rejected”. Features “confirmed” by *Boruta* were deemed available for further analyses. Second, if two features are highly correlated among themselves, they provide redundant information in regards to the outcome, so a filter method that is based on Spearman’s correlation was conducted to further reduce the dimensionality. A correlation matrix was created with all the *Boruta* “confirmed” features. Highly correlated features (Spearman’s correlation coefficients > 0.75) were identified and removed, after which the final selected features were used to construct the nomogram.

### Development of the Nomogram

Data from the training cohort was used to develop the nomogram. First, univariate and multivariate logistic regression analyses were performed to determine the independent predictor of breast malignancy. Candidate factors included results from feature selection and patient age. Non-linear relationships between continuous predictors and malignancy risk were assessed, and continuous predictors with significant non-linearity were transformed into categorical variables using restricted cubic splines (RCS) with three knots (25). Factors with *p* value < 0.2 in univariable analyses were entered in multivariable analyses, which were conducted using stepwise logistic regression with backward elimination at an α level of 0.05.

Basic assumptions that must be met for logistic regression model include linearity between each predictor and outcome, absence of high multicollinearity among predictors, and no strongly influential outliers. To ensure that all logistic regression assumptions were valid, multicollinearity and influential outliers were also assessed. Multicollinearity was estimated by variance inflation factor (VIF), VIF values greater than 4 were an indication of multicollinearity problems (26). Influential outliers were checked by visualizing Cook’s distance (27) and standardized residuals, cases with Cook’s distance of ≥ 0.05 or standardized residuals of ≥3 (28) were considered to be outliers that had unduly large influences on the results. Therefore, they were further analyzed to determine whether they could be excluded from the model.

Finally, based on findings from the above logistic regression analysis, a web-based interactive nomogram was formulated.

### Validation of the Nomogram

Internal and external validations were used to measure the nomogram’s performance. The training cohort was used for internal validation while the two validation cohorts were used for external validation.

Performance was assessed using tests for discrimination, calibration, and clinical usefulness. The discriminative capacity was evaluated *via* receiver operating characteristic (ROC) curve analysis and measured by the area under the receiver operating characteristic curve (AUC). Calibration performance was visually assessed using a calibration plot (29), representing the agreement between observed outcomes and predicted probabilities. The Hosmer–Lemeshow test (30) was performed to assess goodness-of-fit. Finally, decision curve analysis (31, 32) was used to evaluate the clinical benefit of the nomogram by quantifying net benefits at different threshold probabilities.

### Data Analysis

All data analyses and plots were performed and established using R Studio software (R version 4.0.2). The reported statistical significance levels were all two-sided, with *p* value < 0.05 being the threshold for significance, unless otherwise indicated.

Normality of distributions of continuous variables was assessed using the Shapiro–Wilk test. Continuous variables are expressed as medians and ranges, while categorical variables are shown as numbers and percentages. Comparisons between groups were performed using the Chi-square test for categorical variables, while the Wilcoxon test or Student’s t-test were used for continuous variables.

Sample size estimation for reliability analysis was performed using “ICC Sample Size” in R. Inter-rater and intro-rater reliability was calculated using a single-rating, absolute-agreement, 2-way random-effects correlation coefficients (ICCs, model A,1). Reliability was classified as excellent (ICC > 0.90), good (ICC = 0.76–0.90), moderate (ICC = 0.51–0.75), or poor (ICC < 0.50) (33).

Feature selection was performed using “*Boruta*” in R. Correlations between any two morphological features were measured by Spearman rank correlation coefficient while “ggcorrplot” in R was used for visualization of the correlation matrix. The 3D scatter plots were produced using “plotly” in R.

The “glm” function in R was used to fit the multivariate logistic regression model. Regression diagnostics were used to assess the validity of the model, RCS analyses were performed using the “rms” package, multicollinearity was tested by calculating VIF using the “car” package, while influential outliers were graphly inspected by Cook’s distance using the “broom” package. The “rms” and “DynNom” packages were used to develop the nomogram and the web-based calculator, respectively.

Performance evaluation, including visualizations of ROC, Calibration, and DCA, were generated with R packages “ggplot2”, “Caret” and “rmda”. The “pROC” package was used to measure AUCs and conduct the Delong test, while the “ResourceSelection” package was used for the Hosmer–Lemeshow test.

## Results

### Basic Information

**Figure 2:**
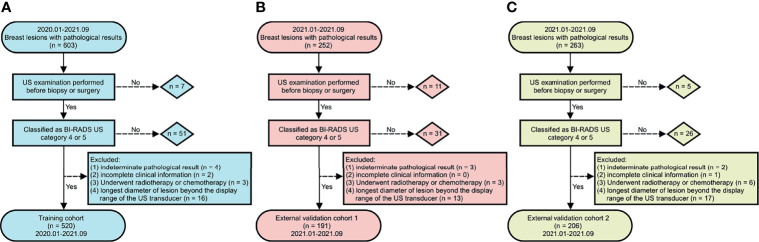
Flow chart of study population. **(A)** training cohort, **(B)** external validation cohort 1, and **(C)** external validation cohort 2.

The flow chart of the study population is presented in **Figure 2**. In total, 917 breast lesions from 917 women were assessed in the study. The final histopathological diagnoses revealed 502 (54.74%) benign and 415 (45.26%) malignant lesions. The training cohort had 520 patients, the external validation cohort from Center B (cohort 1) had 191 patients, while the external validation cohort from Center C (cohort 2) had 206 patients. **Table 1** presents an overview of demographics and baseline characteristics for these study cohorts. While the cohorts did not show significant differences in patients’ age and maximum diameters of lesions, there were significant differences with regards to proportions of benign and malignant lesions among the cohorts. As shown in **Table 1**, the predominant histology of malignant lesions for each cohort was invasive ductal carcinoma, the majority of benign lesions in this study had a breast tumor histology described as fibroadenoma, followed by mammary adenosis.

**Table 1:** Comparisons of patient demographics and baseline characteristics in the training and validation cohorts.

	Training Cohort (n = 520)	External Validation Cohorts		*P*-value
		Cohort 1 (n = 191)	Cohort 2 (n = 206)	
**Age, years (Md (IQR))**	51.5 (44.0, 58.0)	52.0 (45.0, 60.0)	54.0 (46.0, 58.0)	0.430
**Maximum diameter (n,%)**				0.786
<10 mm	34 (6.54)	15 (7.85)	9 (4.37)	
10-20 mm	201 (38.65)	71 (37.17)	77 (37.38)	
20-30 mm	150 (28.85)	59 (30.89)	68 (33.01)	
≥30 mm	135 (25.96)	46 (24.09)	52 (25.24)	
**Pathological outcome (n,%)**				0.010
Benign lesions	295 (56.73)	113 (59.16)	94 (45.63)	
Malignant lesions	225 (43.27)	78 (40.84)	112 (54.37)	
**Histologic subtypes (n,%), Benign**				
Fibroadenoma	135 (25.96)	52 (27.23)	44 (21.36)	
Mammary adenosis	97 (18.65)	42 (21.99)	41 (19.90)	
Intraductal papilloma	53 (10.19)	17 (8.90)	7 (3.40)	
Mastitis	8 (1.54)	2 (1.05)	2 (0.97)	
Benign phyllodes tumor	2 (0.38)	0 (0.00)	0 (0.00)	
**Histologic subtype (n,%), Malignant**				
Invasive ductal carcinoma	192 (36.92)	75 (39.27)	102 (49.51)	
Ductal carcinoma in situ	22 (4.23)	2 (1.05)	4 (1.94)	
Mucous carcinoma	6 (1.15)	0 (0.0)	2 (0.97)	
Invasive lobular carcinoma	3 (0.58%)	1 (0.52)	2 (0.97)	
Solid papillary carcinoma	2 (0.38%)	0 (0.0)	1 (0.49)	

### Morphometric Features

**Figure 3:**
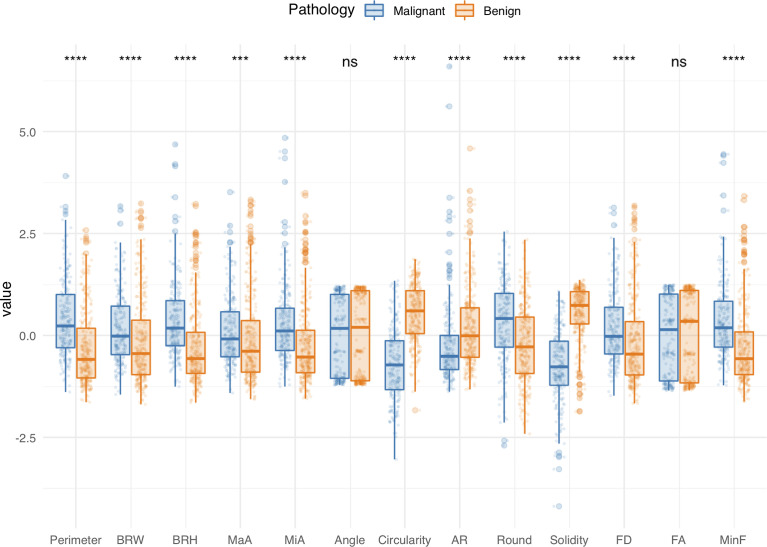
Comparisons of morphological features between benign and malignant groups in the training cohort. Boxplots grouped by pathology show median (horizontal bars), IQR (boxes), and 95% CI (whiskers). Raw data points for each group are shown at the bottom of each box plot. Data were normalized and centered by Z-score transformation to appear on the same scale. Statistical analysis was performed using the Wilcoxon rank-sum test (all features except Round) and Student’s t test (Round). ***p < 0.001, ****p < 0.0001, ns, not significant.

All of the morphometric feature data are available on GitHub (see *Data Availability*). **Figure 3** shows the findings obtained from preliminary analysis of morphometric features in the training cohort. Apart from Angle and FA, the other morphometric features were significantly different between benign and malignant groups. Perimeter, BRW, BRH, MaA, MiA, Round, FD, and MinF values of benign lesions were significantly lower than those of malignant lesions (*p* < 0.001), while Circularity, AR, and Solidity were significantly higher than those of malignant lesions (*p* < 0.001). Morphometric features of the validation cohorts are presented in **Supplementary Figures 1, 2**, respectively.

### Reliability of Morphological Feature Measurements

Inter- and intra-rater reliability of measurement as estimated by the ICC was good or excellent for all morphometric features, apart from inter-rater reliability of Circularity, which was moderate. The ICCs for all morphological features are shown in **Supplement Material Page 6, 7**.

### Feature Selection

**Figure 4:**
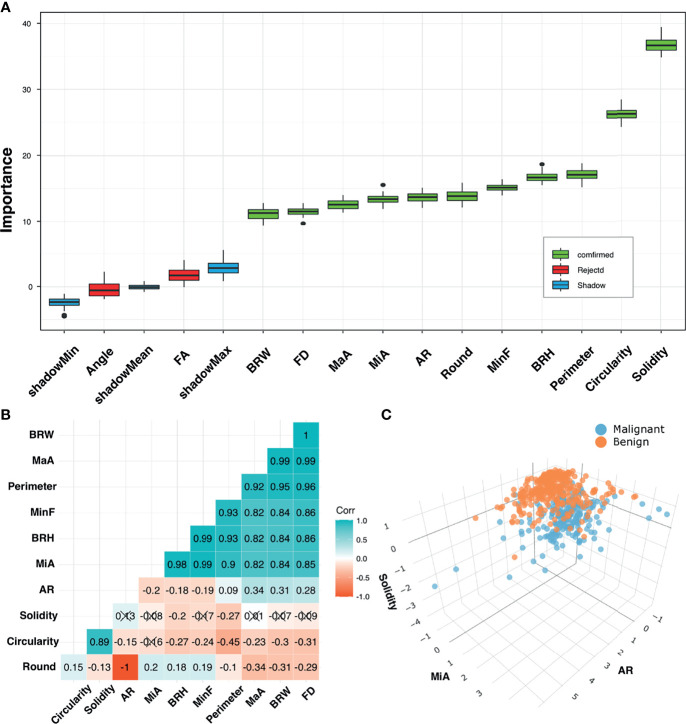
Feature selection. **(A)** Selection of relevant morphometric features for discrimination between benign and malignant groups in the training cohort using the *Boruta* algorithm. Boxplots of features were sorted by increasing importance according to Z-scores. Blue boxes (Shadow) correspond to minimal, mean, and maximal importance, calculated from randomly permuted features. **(B)** Correlation matrix plot shows pairwise positively stronger correlations (blue) or negatively stronger correlations (red). Non-significant correlations (p > 0.05) are marked with a cross. **(C)** 3D scatter plots for final selected feature combinations displaying separations of benign and malignant groups.

**Figure 4** shows the feature selection results. The *Boruta* algorithm and Spearman’s correlation analysis identified 3 features as important and less correlated variables. The results are presented by interactive three-dimensional scatter plots (https://chart-studio.plotly.com/~qingling.go/5/#plot). The selected features were Solidity, AR, and MiA, which were then fed into the nomogram as inputs.

### Development of the Nomogram

#### Univariate and Multivariate Analyses

**Figure 5:**
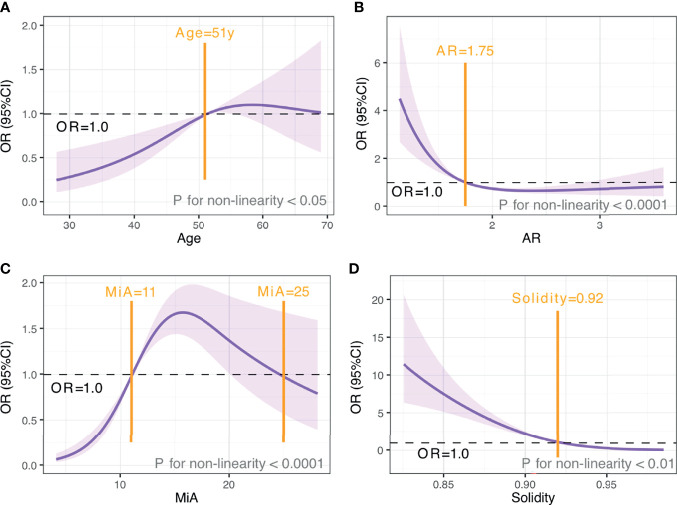
The relationship between age and morphometric features with malignancy risk. OR and 95% CI for age **(A)**, AR **(B),** MiA **(C)**, and Solidity **(D).** The analyses used restricted cubic splines. Purple shaded areas, 95% CIs. Black horizontal dotted line, OR=1.00. Yellow vertical solid line, cut-off value.

We used restricted cubic splines to flexibly model and visualize the associations between age and morphometric features with malignancy risk (**Figure 5**). Since all these variables showed non-linear relationships with malignancy risk, we transformed them into categorical variables. The points where odds ratio (OR) ≈ 1.00 were chosen as the cutoff value according to the trend and knots position of the RCS curve; more importantly, these cut points showed the best performance in the following model fit test. As shown in **Figure 5**, for age < 51 years, malignancy risk gradually increased with age, while above 51 years, the risk was relatively flat, reaching the highest at around 59 years and gradually decreasing thereafter. When AR < 1.75 or Solidity < 0.92, malignancy risk decreased sharply and then leveled off. Regarding the strong inverted-U-shaped relationship between MiA and malignancy risk, the plot showed a substantial increase in the risk, which was highest at around 16, and decreased thereafter. After multiple comparisons of model fits, we found that the model with MiA cutoff at 11 and 25 can achieve the smallest Akaike information criterion (AIC), suggesting the best model fit.

**Table 2** shows the results of univariate and multivariate analyses in the training cohorts. Morphometric features (AR, MiA, Solidity, and age) of patients were all identified as independent predictors for breast malignancy (all *p* < 0.05).

**Table 2:** Results of univariate and multivariate analyses for breast malignancy in the training group.

	Univariate analysis			Multivariate analysis		
	OR	95%CI	*P*-value	OR	95%CI	*P*-value
**Age**						
>51	Ref.			Ref.		
≤51	0.61	0.43~0.87	0.006	0.618	0.38~0.99	0.048
**AR**						
>1.75	Ref.			Ref.		
≤1.75	2.37	1.66~3.38	<0.001	2.01	1.24~3.26	0.005
**MiA**						
<11	Ref.			Ref.		
11-25	3.24	2.24~4.68	<0.001	3.83	2.32~6.46	< 0.001
≥25	1.95	0.78~4.89	0.156	8.02	2.65~23.83	< 0.001
**Solidity**						
>0.92	Ref.			Ref.		
≤0.92	20.28	12.83~32.06	<0.001	25.81	15.47~44.80	< 0.001

Factors associated with dependent variables with p < 0.2 in univariate analysis were entered into the logistic backward step-wise multivariate model.

#### Logistics Regression Diagnostics

(1) Nonlinear relationships. Nonlinear relationships between predictors and pathological outcomes were resolved by RCS analyses. (2) Multicollinearity. All VIF values are below the threshold value of 2 (Age, VIF = 1.02; AR, VIF = 1.04, MiA, VIF = 1.23, Solidity, VIF = 1.20), indicating the absence of collinearity among predictors. (3) Influential outliers. As shown in **Supplementary Figures 3, 4**, no outliers were identified by Cook’s distance or standardized residuals. The above findings indicate that all logistic regression assumptions for our model were met.

#### Nomogram and Web-Based Calculator

**Figure 6:**
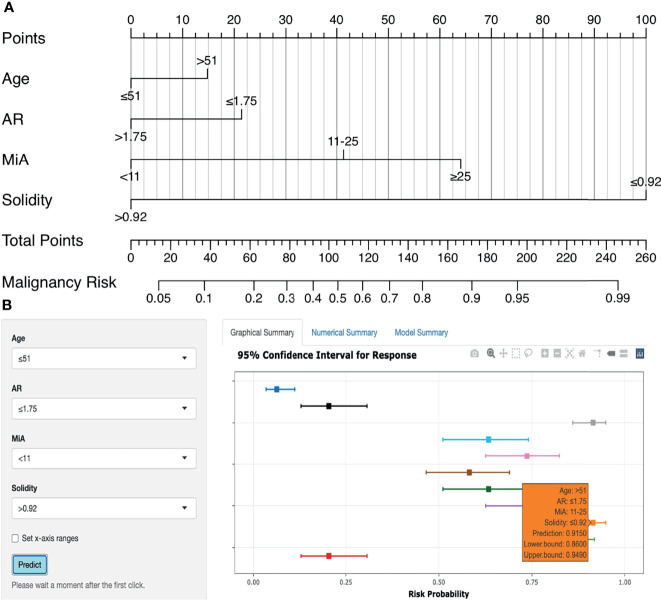
Nomogram and online risk calculator. **(A)** Nomogram based on US morphometric features. Applications of the nomogram were exemplified in **Supplementary Figure 5**. **(B)** The online calculator application version of the nomogram.

**Figure 6A** shows the nomogram for predicting breast malignancy based on independent risk factors, including US morphometric features AR, MiA, and Solidity. Based on the above nomogram, we established an online risk calculator to facilitate the use of the nomogram by clinicians, which can be freely accessed at https://qingling.shinyapps.io/DynNomapp/(**Figure 6B**). Using quantitative values of lesion morphological features, the calculator can individually predict the risk of breast malignancy. For instance, for patients aged > 51 years whose AR ≤ 1.75, MiA 11-25 and Solidity ≤ 0.92, the risk probability of malignancy was approximately 91.5% (95% CI 86.0–94.9%).

### Validation of the Nomogram

#### Discrimination

**Figure 7:**
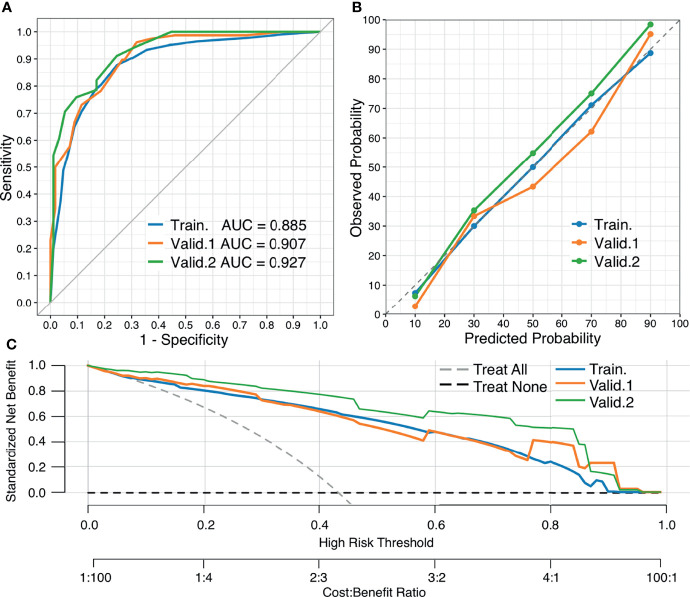
Performance of the nomogram. **(A)** ROC curves of the nomogram in the training and external validation cohorts, respectively. **(B)** Calibration curves of the nomogram, which depict calibration of the nomogram in terms of agreement between the predicted risk of breast malignancy and observed outcomes. The diagonal dotted line denotes a perfect prediction, the closer the calibration curve fit is to the diagonal line, the better the predictive accuracy of the nomogram. **(C)** DCA curves of the nomogram. The gray and black dotted lines represent the hypothesis that all patients had a diagnosis of breast malignancy (“treat all”) and that no patients had a diagnosis of breast malignancy (“treat none”), respectively. X-axis indicates the threshold probability for pathological outcomes while the Y-axis indicates the standardized net benefit for a given threshold probability.

The AUCs of the nomogram in the training and validation cohorts were 0.885, 0.907, and 0.927, respectively (**Figure 7A**). There were no significant differences in AUCs between any two cohorts (DeLong test, p > 0.05 for each comparison, **Supplementary Table 2**). Therefore, our nomogram performed well in all the training and validation cohorts.

#### Calibration

Calibration curves of the nomogram are close to the diagonal line in the training and validation cohorts, demonstrating that the predictive probability has good agreement with observed outcomes (**Figure 7B**). The Hosmer–Lemeshow test yielded a non-significant statistic (p = 0.94), indicating a good fit.

#### Clinical Utility

DCA curves of the training and validation cohorts revealed clinical usefulness of the nomogram (**Figure 7C**). From this figure, it can be seen that in all the training and validation cohorts, the nomogram has a higher net benefit than both “treat all” and “treat none” across the range of threshold probabilities 10-90%, indicating that the nomogram was clinically useful, that is, the nomogram would improve patient outcome irrespective of patient or doctor preference for a reasonable threshold probability.

## Discussion

In this retrospective multicenter study of 917 patients with breast lesions, we analyzed US morphometric features and developed a simple-to-use nomogram for predicting cancer. The newly developed nomogram performed well, and its predictive value was validated using data from other hospitals in a different geographic region. Our nomogram has three ultrasonic morphometric features that are easy to generate using ImageJ software and that radiologists can easily understand and interpret. The nomogram can adapt to different ultrasonic instruments and settings, and it has a high generalization ability and practicality. To make the nomogram user-friendly, we have availed it as a free web-based calculator. Consequently, the nomogram developed in this study will potentially be a valuable tool in clinical practices.

As precision medicine advances, the nomogram, which can provide an individual patient with a quantitative risk assessment of a particular outcome by a graphical interface, has been proposed as a simple and reliable means to improve disease prediction or prognosis (34, 35). Several US-based nomograms for predicting cancer risk or prognosis have been reported in the literature; all of these nomograms demonstrated high predictive performance with AUC = 0.747–0.951. Some of the nomograms were based on subjective evaluations using qualitative descriptors (e.g., spiculated, rounded, microcalcification, etc.), which are highly dependent on the level of expertise and experience and can suffer from a large intra- and inter-observer variability (36–39). However, other nomograms were based on quantitative methods such as radiomics, which can objectively describe tumor phenotypes using numerical features extracted from radiological images (14–16). These features, which are mainly related to tumor size, shape, texture, and intensity, provide a comprehensive tumor characterization. In this scenario, quantitative evaluation of US images is a natural consequence of the path towards personalized medicine.

The present study is based on quantitative features, and the performance of our nomogram was very comparable to that of the preceding studies, with a few notable differences. The first difference is that we only used the morphometric features to construct the nomogram, which was due to the following reasons. As a diagnostic or prognostic tool, a nomogram must be practical and generalizable in clinical settings. However, the reproducibility of quantitative features based on image pixels, including textural features, intensity-based features, and wavelet-based features, is affected by image preprocessing to variable degrees (19, 40, 41). Recently, Lee SE et al. found that the radiomics of textural features differed depending on the type of US machine (42). Previous literature has also associated the measurement of textural parameters with nonlinear variations in ultrasonic system settings such as time-gain compensation, total gain, and focal depth (43). Overall, these studies consistently indicated that due to variations in acquisition modes, parameter selections, or implementation procedures, the features derived from the pixel gray-level statistics in terms of intensity and spatial distribution have greater variability, particularly for US technique, which is more operator- and system-dependent. In contrast, the morphometric features characterize the shape and contour of a lesion and are essentially independent of the system settings and machines (22, 44). More importantly, the morphologic traits of breast cancer are associated with histological findings (21, 45, 46), which can provide valuable information for deriving robust multidisciplinary models (47). In this study, we found that most of the morphometric features differed significantly between benign and malignant groups, and the contributions of the selected features to the prediction model were as follows: Solidity > Circularity > Minor Axis. These results confirmed the association between ultrasonography morphologic features and histopathological findings.

The second difference is that in previous studies, the images from the US were almost entirely collected in one specific US machine and system (14–16), whereas in this study, the training and validation data were both pooled from different US machines and systems. Moreover, the US transducers used for imaging had different transmission frequencies, which is more congruent with the actual clinical settings and a significant strength of this study. The results with the external validation cohorts strengthened the predictive potential of the model, increasing our confidence in the robustness and generality of the novel nomogram. Furthermore, we built a web-based calculator with user-friendly digital interfaces to display the nomogram, which makes risk assessment easier. The user inputs the details of the lesion on the web page, and the probability of breast malignancy is calculated automatically for the patient.

Finally, when compared to other quantification-based nomograms (14–17), the predictor variables in our nomogram are easily accessed and interpreted. In general, lack of interpretability is one of the major barriers to successful translation of predictive models from research to clinical practice, particularly for data-driven precision medicine (20). From a clinical perspective, interpretability is critical for winning the trust of physicians, developing a robust decision-making system, and overcoming regulatory concerns (48). For example, it is difficult for radiomics practitioners to interpret first-order entropy or grey level co-occurrence matrix features and to assign biological meaning to them (49). Physicians must be able to interpret the nomogram model and identify the predictors separately for rejected and accepted outcomes, and decide on the subsequent treatment protocols (50). The morphometric features in our nomogram are relatively non-abstract and can be considered an extension of the analytical thinking of a radiologist. This assists radiologists in understanding the decision process of the nomogram and facilitates doctor–patient communication. Furthermore, the morphometric features are relatively easy to retrieve *via* the interactive freeware ImageJ, without the need to run scripts from the command line.

This study has several limitations that are worth mentioning. First, this was a retrospective study, which had inherent biases. Therefore, larger, high-quality prospective studies should be conducted in the future. Second, the distribution of pathological subtypes of breast cancer included in this study was unbalanced, especially for the specific pathological types such as mucinous or medullary breast cancer. In addition, the sample size was relatively small and the specific pathological types had different histological substrates that manifest as different imaging features on US (51, 52), which may have resulted in bias. Third, large dimension lesions were not included in this study, which could have caused spectrum bias in patient selection. Finally, accurate segmentation is necessary for extracting quantitative features from a tumor (53). Although the features extracted using manual segmentation in this study showed high inter-observer and intra-observer reliability, the process was relatively time-consuming when compared to automatic segmentation. These limitations highlight the need for additional research to potentially improve model performance.

## Conclusions

In this multicentric study, we developed an interpretable and simple-to-use dynamic nomogram to quantify the probability of breast malignancy based on US morphometrics. The nomogram demonstrated good discrimination performance between malignant and benign lesions, as well as good calibration and clinical usefulness. Moreover, the nomogram showed high generalization capabilities, suggesting that it may be used in clinical practice as a tool to guide personalized treatment. Our findings show that quantitative morphometric features from different ultrasound machines and systems can be used as imaging surrogate biomarkers for the development of robust and reproducible quantitative ultrasound dynamic models in breast cancer research.

## Data Availability Statement

The datasets presented in this study can be found in online repositories. The names of the repository/repositories and accession number(s) can be found below: https://github.com/QinglingGo/BUS-Morphometric-Datasets.

## Ethics Statement

The First Affiliated Hospital of Wannan Medical College Review Board, Shandong Provincial Third Hospital Review Board and Linyi People’s Hospital Review Board approved this retrospective study. Written informed consent was waived due to the use of retrospective, de-identified information from the image database.

## Author Contributions

QinglingZ: conceptualization, data curation, supervision, data analysis, and writing (original draft). QingluZ and TL: data curation, interpretation, and writing (review and editing). TB, QL, and YY: US morphometric data collection. All authors contributed to the article and approved the submitted version.

## Conflict of Interest

The authors declare that the research was conducted in the absence of any commercial or financial relationships that could be construed as a potential conflict of interest.

## Publisher’s Note

All claims expressed in this article are solely those of the authors and do not necessarily represent those of their affiliated organizations, or those of the publisher, the editors and the reviewers. Any product that may be evaluated in this article, or claim that may be made by its manufacturer, is not guaranteed or endorsed by the publisher.

## References

[1] Sung H Ferlay J Siegel RL Laversanne M Soerjomataram I Jemal A. Global Cancer Statistics 2020: Globocan Estimates of Incidence and Mortality Worldwide for 36 Cancers in 185 Countries. CA Cancer J Clin (2021) 71(3):209–49. doi: 10.3322/caac.21660 33538338

[2] Basu P Ponti A Anttila A Ronco G Senore C Vale DB. Status of Implementation and Organization of Cancer Screening in the European Union Member States-Summary Results From the Second European Screening Report. Int J Cancer (2018) 142(1):44–56. doi: 10.1002/ijc.31043 28940326

[3] Hogben RK. Screening for Breast Cancer in England: A Review. Curr Opin Obstet Gynecol (2008) 20(6):545–9. doi: 10.1097/GCO.0b013e3283186fab 18989129

[4] Evans A Trimboli RM Athanasiou A Balleyguier C Baltzer PA Bick U. Breast Ultrasound: Recommendations for Information to Women and Referring Physicians by the European Society of Breast Imaging. Insights Imaging (2018) 9(4):449–61. doi: 10.1007/s13244-018-0636-z PMC6108964 30094592

[5] Pan B Yao R Zhu QL Wang CJ You SS Zhang J. Clinicopathological Characteristics and Long-Term Prognosis of Screening Detected Non-Palpable Breast Cancer by Ultrasound in Hospital-Based Chinese Population (2001-2014). Oncotarget (2016) 7(47):76840–51. doi: 10.18632/oncotarget.12319 PMC5363553 27689334

[6] Berg WA Bandos AI Mendelson EB Lehrer D Jong RA Pisano ED. Ultrasound as the Primary Screening Test for Breast Cancer: Analysis From Acrin 6666. J Natl Cancer Inst (2016) 108(4):djv367. doi: 10.1093/jnci/djv367 26712110 PMC5943835

[7] Bozzini A Nicosia L Pruneri G Maisonneuve P Meneghetti L Renne G. Clinical Performance of Contrast-Enhanced Spectral Mammography in Pre-Surgical Evaluation of Breast Malignant Lesions in Dense Breasts: A Single Center Study. Breast Cancer Res Treat (2020) 184(3):723–31. doi: 10.1007/s10549-020-05881-2 PMC7655556 32860166

[8] Mendelson EB Böhm-Vélez M Berg WA D'Orsi CJ Sickles EA. ACR BI-RADS©Ultrasound. In: ACR BI-RADS©Atlas, Breast Imaging Reporting and Data System. Reston, VA: American College of Radiology (2013).

[9] Zou X Wang J Lan X Lin Q Han F Liu L. Assessment of Diagnostic Accuracy and Efficiency of Categories 4 and 5 of the Second Edition of the Bi-Rads Ultrasound Lexicon in Diagnosing Breast Lesions. Ultrasound Med Biol (2016) 42(9):2065–71. doi: 10.1016/j.ultrasmedbio.2016.04.020 27262521

[10] Stavros AT Freitas AG deMello GGN Barke L McDonald D Kaske T. Ultrasound Positive Predictive Values by Bi-Rads Categories 3-5 for Solid Masses: An Independent Reader Study. Eur Radiol (2017) 27(10):4307–15. doi: 10.1007/s00330-017-4835-7 28396996

[11] Spinelli Varella MA Teixeira da Cruz J Rauber A Varella IS Fleck JF Moreira LF. Role of Bi-Rads Ultrasound Subcategories 4a to 4c in Predicting Breast Cancer. Clin Breast Cancer (2018) 18(4):e507–e11. doi: 10.1016/j.clbc.2017.09.002 29066139

[12] Kim YR Kim HS Kim HW. Are Irregular Hypoechoic Breast Masses on Ultrasound Always Malignancies?: A Pictorial Essay. Korean J Radiol (2015) 16(6):1266–75. doi: 10.3348/kjr.2015.16.6.1266 PMC4644748 26576116

[13] Nicosia L Addante F Bozzini AC Latronico A Montesano M Meneghetti L. Evaluation of Computer-Aided Diagnosis in Breast Ultrasonography: Improvement in Diagnostic Performance of Inexperienced Radiologists. Clin Imaging (2022) 82:150–5. doi: 10.1016/j.clinimag.2021.11.006 34826773

[14] Luo WQ Huang QX Huang XW Hu HT Zeng FQ Wang W. Predicting Breast Cancer in Breast Imaging Reporting and Data System (Bi-Rads) Ultrasound Category 4 or 5 Lesions: A Nomogram Combining Radiomics and Bi-Rads. Sci Rep (2019) 9(1):11921. doi: 10.1038/s41598-019-48488-4 31417138 PMC6695380

[15] Qiu X Jiang Y Zhao Q Yan C Huang M Jiang T. Could Ultrasound-Based Radiomics Noninvasively Predict Axillary Lymph Node Metastasis in Breast Cancer? J Ultrasound Med (2020) 39(10):1897–905. doi: 10.1002/jum.15294 PMC7540260 32329142

[16] Xiong L Chen H Tang X Chen B Jiang X Liu L. Ultrasound-Based Radiomics Analysis for Predicting Disease-Free Survival of Invasive Breast Cancer. Front Oncol (2021) 11:621993. doi: 10.3389/fonc.2021.621993 33996546 PMC8117589

[17] Zha HL Zong M Liu XP Pan JZ Wang H Gong HY. Preoperative Ultrasound-Based Radiomics Score Can Improve the Accuracy of the Memorial Sloan Kettering Cancer Center Nomogram for Predicting Sentinel Lymph Node Metastasis in Breast Cancer. Eur J Radiol (2021) 135:109512. doi: 10.1016/j.ejrad.2020.109512 33429302

[18] Lambin P Leijenaar RTH Deist TM Peerlings J de Jong EEC van Timmeren J. Radiomics: The Bridge Between Medical Imaging and Personalized Medicine. Nat Rev Clin Oncol (2017) 14(12):749–62. doi: 10.1038/nrclinonc.2017.141 28975929

[19] Traverso A Wee L Dekker A Gillies R. Repeatability and Reproducibility of Radiomic Features: A Systematic Review. Int J Radiat Oncol Biol Phys (2018) 102(4):1143–58. doi: 10.1016/j.ijrobp.2018.05.053 PMC6690209 30170872

[20] Vuong D Tanadini-Lang S Wu Z Marks R Unkelbach J Hillinger S. Radiomics Feature Activation Maps as a New Tool for Signature Interpretability. Front Oncol (2020) 10:578895. doi: 10.3389/fonc.2020.578895 33364192 PMC7753181

[21] Kim SH Seo BK Lee J Kim SJ Cho KR Lee KY. Correlation of Ultrasound Findings With Histology, Tumor Grade, and Biological Markers in Breast Cancer. Acta Oncol (2008) 47(8):1531–8. doi: 10.1080/02841860801971413 18607848

[22] El-Azizy ARM Salaheldien M Rushdi MA Gewefel H Mahmoud AM. Morphological Characterization of Breast Tumors Using Conventional B-Mode Ultrasound Images. Annu Int Conf IEEE Eng Med Biol Soc (2019) 2019:6620–3. doi: 10.1109/EMBC.2019.8857438 31947359

[23] Zou GY. Sample Size Formulas for Estimating Intraclass Correlation Coefficients With Precision and Assurance. Stat Med (2012) 31(29):3972–81. doi: 10.1002/sim.5466 22764084

[24] Kursa MB Rudnicki WR. Feature Selection With the *Boruta* Package. J Stat Softw (2010) 36(11):1–13. doi: 10.18637/jss.v036.i11

[25] Durrleman S Simon R. Flexible Regression Models With Cubic Splines. Stat Med (1989) 8(5):551–61. doi: 10.1002/sim.4780080504 2657958

[26] Kirshner JJ Heckler CE Janelsins MC Dakhil SR Hopkins JO Coles C. Prevention of Pegfilgrastim-Induced Bone Pain: A Phase Iii Double-Blind Placebo-Controlled Randomized Clinical Trial of the University of Rochester Cancer Center Clinical Community Oncology Program Research Base. J Clin Oncol (2012) 30(16):1974–9. doi: 10.1200/JCO.2011.37.8364 PMC3383174 22508813

[27] Cook RD. Detection of Influential Observation in Linear Regression. Technometrics (1977) 19(1):15–8. doi: 10.1080/00401706.1977.10489493

[28] Sorge RE Mapplebeck JC Rosen S Beggs S Taves S Alexander JK. Different Immune Cells Mediate Mechanical Pain Hypersensitivity in Male and Female Mice. Nat Neurosci (2015) 18(8):1081–3. doi: 10.1038/nn.4053 PMC4772157 26120961

[29] Steyerberg EW Vickers AJ Cook NR Gerds T Gonen M Obuchowski N. Assessing the Performance of Prediction Models: A Framework for Traditional and Novel Measures. Epidemiology (2010) 21(1):128–38. doi: 10.1097/EDE.0b013e3181c30fb2 PMC3575184 20010215

[30] Hosmer DW Lemeshow S. Goodness-Of-Fit Tests for the Multiple Logistic Regression Model. Commun Stat-Theor M (1980) 9(10):1043–69. doi: 10.1080/03610928008827941

[31] Vickers AJ Elkin EB. Decision Curve Analysis: A Novel Method for Evaluating Prediction Models. Med Decis Making (2006) 26(6):565–74. doi: 10.1177/0272989X06295361 PMC2577036 17099194

[32] Vickers AJ van Calster B Steyerberg EW. A Simple, Step-By-Step Guide to Interpreting Decision Curve Analysis. Diagn Progn Res (2019) 3:18. doi: 10.1186/s41512-019-0064-7 31592444 PMC6777022

[33] Koo TK Li MY. A Guideline of Selecting and Reporting Intraclass Correlation Coefficients for Reliability Research. J Chiropr Med (2016) 15(2):155–63. doi: 10.1016/j.jcm.2016.02.012 PMC4913118 27330520

[34] Balachandran VP Gonen M Smith JJ DeMatteo RP. Nomograms in Oncology: More Than Meets the Eye. Lancet Oncol (2015) 16(4):173–80. doi: 10.1016/S1470-2045(14)71116-7 PMC4465353 25846097

[35] Martinez-Perez C Turnbull AK Ekatah GE Arthur LM Sims AH Thomas JS. Current Treatment Trends and the Need for Better Predictive Tools in the Management of Ductal Carcinoma in Situ of the Breast. Cancer Treat Rev (2017) 55:163–72. doi: 10.1016/j.ctrv.2017.03.009 28402908

[36] Zhou P Jin C Lu J Xu L Zhu X Lian Q. Modified Model for Diagnosing Breast Imaging Reporting and Data System Category 3 to 5 Breast Lesions: Retrospective Analysis and Nomogram Development. J Ultrasound Med (2021) 40(1):151–61. doi: 10.1002/jum.15385 32681744

[37] Niu Z Tian JW Ran HT Ren WD Chang C Yuan JJ. Risk-Predicted Dual Nomograms Consisting of Clinical and Ultrasound Factors for Downgrading Bi-Rads Category 4a Breast Lesions - a Multiple Centre Study. J Cancer (2021) 12(1):292–304. doi: 10.7150/jca.51302 33391426 PMC7738830

[38] Liang T Cong S Yi Z Liu J Huang C Shen J. Ultrasound-Based Nomogram for Distinguishing Malignant Tumors From Nodular Sclerosing Adenoses in Solid Breast Lesions. J Ultrasound Med (2021) 40(10):2189–200. doi: 10.1002/jum.15612 33438775

[39] Yang Y Hu Y Shen S Jiang X Gu R Wang H. A New Nomogram for Predicting the Malignant Diagnosis of Breast Imaging Reporting and Data System (Bi-Rads) Ultrasonography Category 4a Lesions in Women With Dense Breast Tissue in the Diagnostic Setting. Quant Imaging Med Surg (2021) 11(7):3005–17. doi: 10.21037/qims-20-1203 PMC8250024 34249630

[40] Yip SS Aerts HJ. Applications and Limitations of Radiomics. Phys Med Biol (2016) 61(13):R150–66. doi: 10.1088/0031-9155/61/13/R150 PMC4927328 27269645

[41] Pesapane F Rotili A Agazzi GM Botta F Raimondi S Penco S. Recent Radiomics Advancements in Breast Cancer: Lessons and Pitfalls for the Next Future. Curr Oncol (2021) 28(4):2351–72. doi: 10.3390/curroncol28040217 PMC8293249 34202321

[42] Lee SE Han K Kwak JY Lee E Kim EK. Radiomics of Us Texture Features in Differential Diagnosis Between Triple-Negative Breast Cancer and Fibroadenoma. Sci Rep (2018) 8(1):13546. doi: 10.1038/s41598-018-31906-4 30202040 PMC6131410

[43] Alvarenga AV Infantosi A Pereira W Azevedo C. Morphometric and Texture Parameters in Distinguishing Breast Tumours on Ultrasound Images. In: Magjarevic R Nagel JH, editors. World Congress on Medical Physics and Biomedical Engineering. Berlin: IFMBE Proceedings (2006). p. 2272–5. doi: 10.1007/978-3-540-36841-0_573

[44] Wu WJ Moon WK. Ultrasound Breast Tumor Image Computer-Aided Diagnosis With Texture and Morphological Features. Acad Radiol (2008) 15(7):873–80. doi: 10.1016/j.acra.2008.01.010 18572123

[45] Malherbe K Bresser P. Association Between Ultrasound Morphologic Features and Histopathological Findings of Lobular Carcinoma. J Med Radiat Sci (2019) 66(3):177–83. doi: 10.1002/jmrs.336 PMC6745349 31472006

[46] Wang H Zhan W Chen W Li Y Chen X Shen K. Sonography With Vertical Orientation Feature Predicts Worse Disease Outcome in Triple Negative Breast Cancer. Breast (2020) 49:33–40. doi: 10.1016/j.breast.2019.10.006 31677531 PMC7375680

[47] Tot T. The Role of Large-Format Histopathology in Assessing Subgross Morphological Prognostic Parameters: A Single Institution Report of 1000 Consecutive Breast Cancer Cases. Int J Breast Cancer (2012) 2012:395415. doi: 10.1155/2012/395415 23150828 PMC3485542

[48] Naik N Madani A Esteva A Keskar NS Press MF Ruderman D. Deep Learning-Enabled Breast Cancer Hormonal Receptor Status Determination From Base-Level H&E Stains. Nat Commun (2020) 11(1):5727. doi: 10.1038/s41467-020-19334-3 33199723 PMC7670411

[49] Parekh VS Jacobs MA. Deep Learning and Radiomics in Precision Medicine. Expert Rev Precis Med Drug Dev (2019) 4(2):59–72. doi: 10.1080/23808993.2019.1585805 31080889 PMC6508888

[50] Das D Ito J Kadowaki T Tsuda K. An Interpretable Machine Learning Model for Diagnosis of Alzheimer's Disease. PeerJ (2019) 7:e6543. doi: 10.7717/peerj.6543 30842909 PMC6398390

[51] Pintican R Duma M Chiorean A Fetica B Badan M Bura V. Mucinous Versus Medullary Breast Carcinoma: Mammography, Ultrasound, and Mri Findings. Clin Radiol (2020) 75(7):483–96. doi: 10.1016/j.crad.2019.12.024 32057415

[52] Chaudhry AR El Khoury M Gotra A Eslami Z Omeroglu A Omeroglu-Altinel G. Imaging Features of Pure and Mixed Forms of Mucinous Breast Carcinoma With Histopathological Correlation. Br J Radiol (2019) 92(1095):20180810. doi: 10.1259/bjr.20180810 30632779 PMC12187173

[53] Thawani R McLane M Beig N Ghose S Prasanna P Velcheti V. Radiomics and Radiogenomics in Lung Cancer: A Review for the Clinician. Lung Cancer (2018) 115:34–41. doi: 10.1016/j.lungcan.2017.10.015 29290259

