# Supplementary material for: Development and External Validation of a Simple-To-Use Dynamic Nomogram for Predicting Breast Malignancy Based on Ultrasound Morphometric Features: A Retrospective Multicenter Study
Source: Front Oncol. 2022 Apr 7;12:868164. doi: 10.3389/fonc.2022.868164 (PMC9021381; doi:10.3389/fonc.2022.868164)
Supplement: Supplementary file 1 [file DataSheet_1.docx]

Supplementary Material

# Supplementary Figures


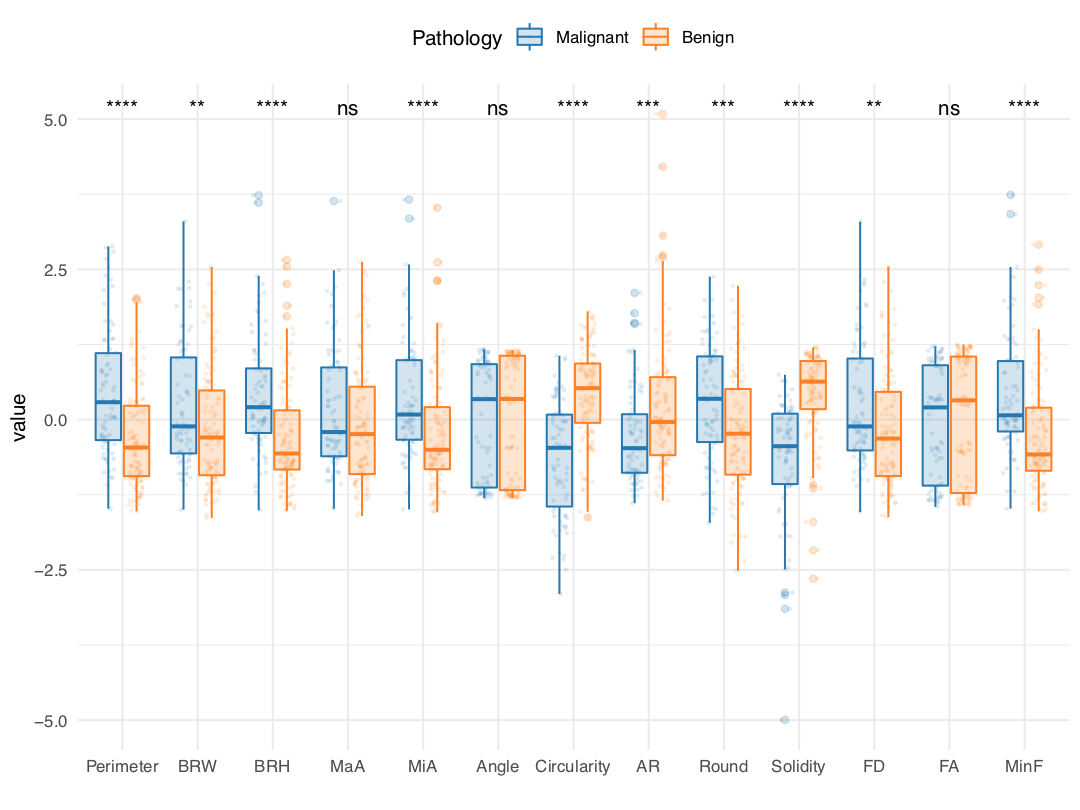


**Supplementary Figure 1.** Comparison of morphological features between benign and malignant groups in the external validation cohort 1. Boxplots grouped by pathology show median (horizontal bars), IQR (boxes), and 95% CI (whiskers). Raw data points of each group are shown at the bottom of each box plot. Data were normalized and centred by Z-score transformation to appear on the same scale. Statistical analysis was performed using Wilcoxon rank-sum test (all features except Round) and Student's t test (Round) ,**p < 0.01, ***p < 0.001, ****p < 0.0001, ns, not significant.


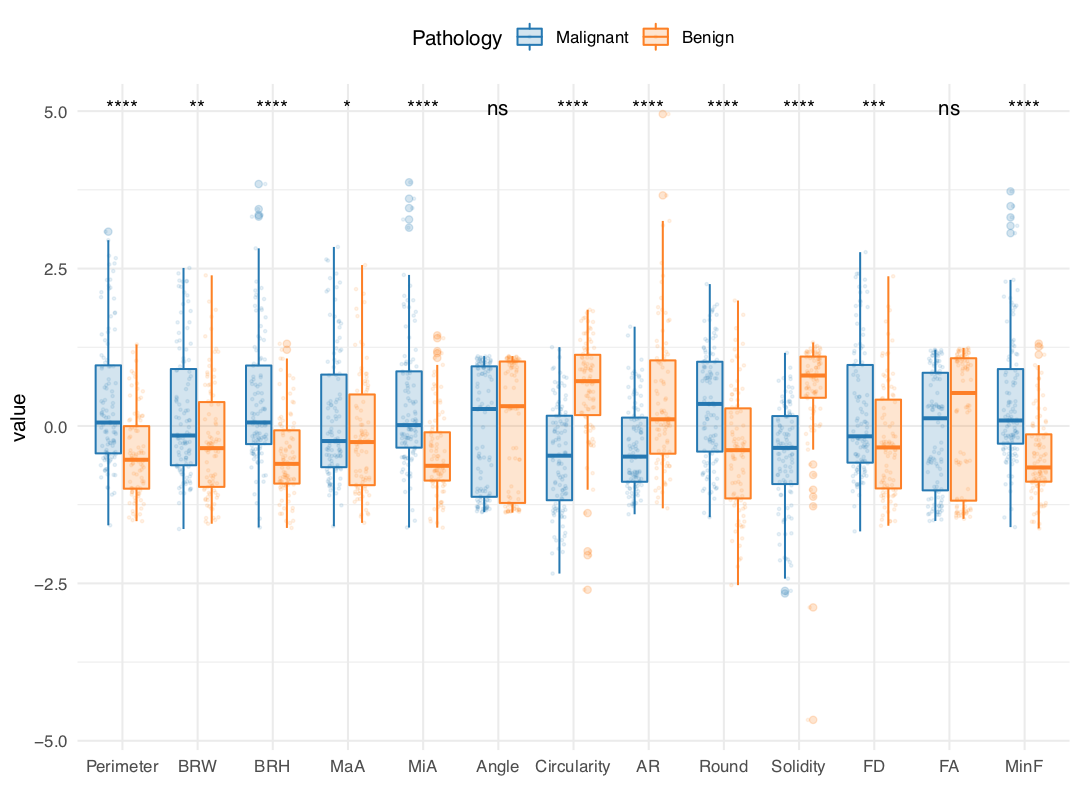


**Supplementary Figure 2.** Comparison of morphological features between benign and malignant groups in the external validation cohort 2. Boxplots grouped by pathology show median (horizontal bars), IQR (boxes), and 95% CI (whiskers). Raw data points of each group are shown at the bottom of each box plot. Data were normalized and centred by Z-score transformation to appear on the same scale. Statistical analysis was performed using Wilcoxon rank-sum test, *p < 0.05, **p < 0.01, ***p < 0.001, ****p < 0.0001, ns, not significant.


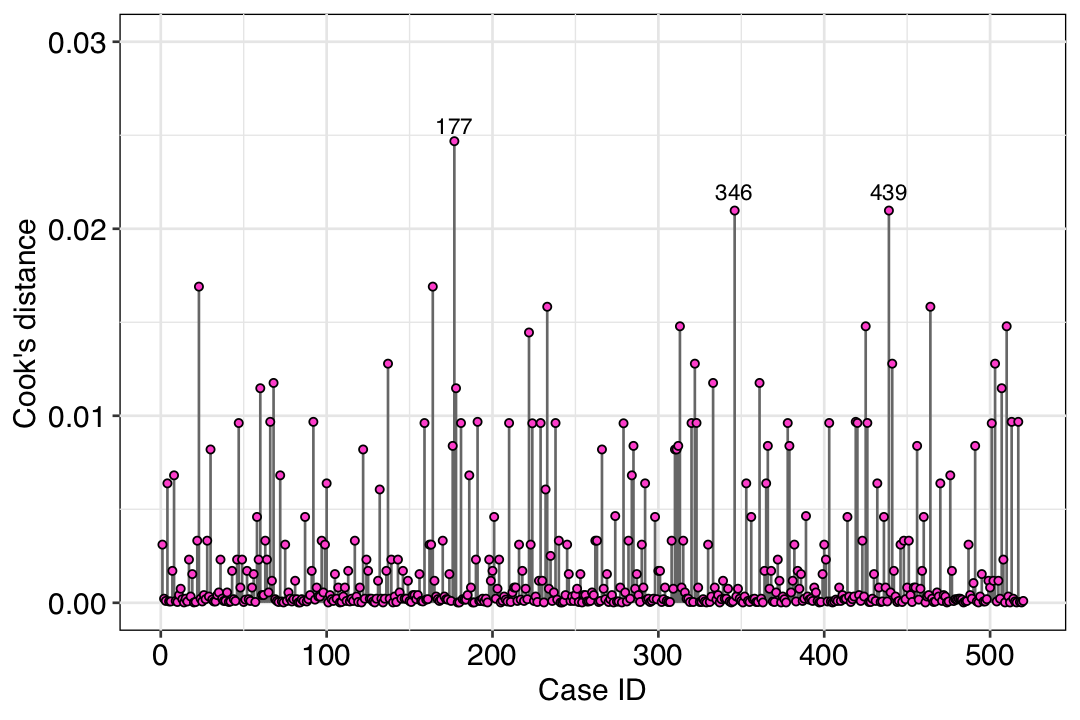


**Supplementary Figure 3.** Validation of logistic regression assumption; check influential outliers by Cook's distance. No influential outliers were detected. The cases with ID 177, 346, and 439 seem to be the most influential observations, but like all other cases in the training cohort, their Cook distances are all < 0.03.


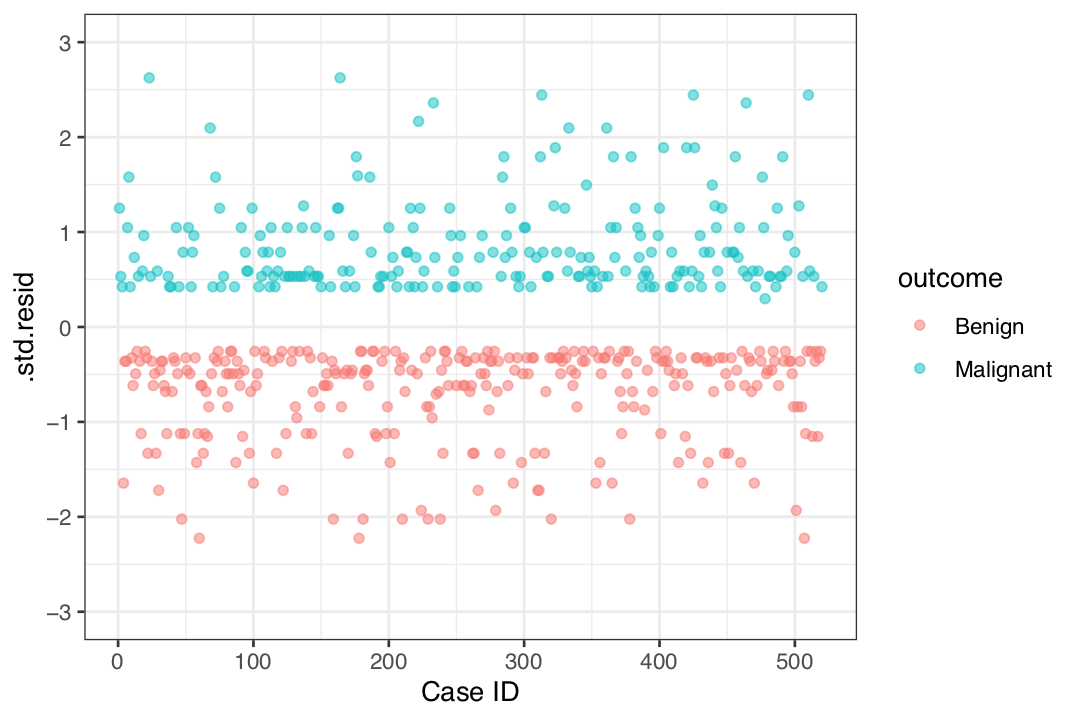


**Supplementary Figure 4.** Validation of logistic regression assumption; check influential outliers by standardized residuals. No influential outliers were detected. The standardized residuals of all cases in the training cohort are < 3.


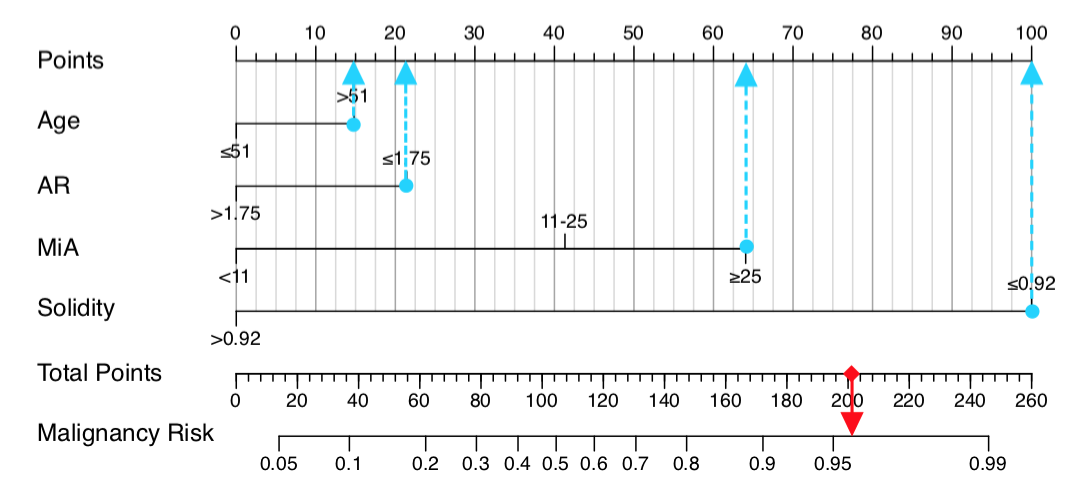


**Supplementary Figure 5.** Applications of Nomogram. For each predictor, read the points assigned on the 0-100 scale at the top and then sum these points. Find the number on the “Total Points” scale and then read the corresponding predictions of breast malignancy risk below it.

# Supplementary Tables

**Supplementary Table 1.** Reliability of ultrasound morphometric features measurement.

|  | **ICC(95% CI)** | |
| --- | --- | --- |
|  | **Inter-rater** | **Intro-rater** |
| Perimeter | 0.943(0.903, 0.966) | 0.983(0.971, 0.990) |
| BRW | 0.978(0.966, 0.986) | 0.985(0.976, 0.991) |
| BRH | 0.973(0.957, 0.983) | 0.988(0.982, 0.992) |
| MaA | 0.988(0.982, 0.993) | 0.991(0.985, 0.994) |
| MiA | 0.973(0.959, 0.983) | 0.989(0.983, 0.993) |
| Angle | 0.834(0.749, 0.892) | 0.942(0.911, 0.962) |
| Circularity | 0.739(0.621, 0.825) | 0.949(0.909, 0.970) |
| AR | 0.853(0.781, 0.903) | 0.979(0.968, 0.987) |
| Round | 0.861(0.791, 0.908) | 0.966(0.947, 0.978) |
| Solidity | 0.784(0.683, 0.856) | 0.962(0.939, 0.976) |
| FD | 0.981(0.970,0.988) | 0.986(0.977, 0.991) |
| FA | 0.842(0.764, 0.895) | 0.888(0.831, 0.927) |
| MinF | 0.973(0.959, 0.983) | 0.989(0.983, 0.993) |

ICC = intraclass correlation coefficient.

**ICC Calculation Summary**

(1) Sample size calculation: Sample size estimation for reliability analysis was performed using the package "ICC Sample Size" in R.

R script:

*library(ICC.Sample.Size)*

*calculateIccSampleSize(p=0.80,p0=0.60,k=2,alpha=0.05,tails=2,power=0.90,by="both",step=0.05)*

**(2) ICC form selection**: single-rating, absolute-agreement, 2-way random-effects correlation coefficients (ICCs, model A,1).

**(3) ICC calculation:** ICC value calculation was performed using the package "icc" in R.

R script example:

*library(irr)*

*valueA_intra <-icc(valueA_intra-ratedata, model = "twoway", type = "agreement",*

*unit = "single", r0 = 0, conf.level = 0.95)*

*valueA_inter <-icc(valueA_inter-ratedata, model = "twoway", type = "agreement",*

*unit = "single", r0 = 0, conf.level = 0.95)*

**(4) Data Availability:** The original datasets used to perform the ICC analyses can be found on GitHub repository (<https://github.com/QinglingGo/BUS-Morphometric-Datasets>).

**Supplementary Table 2.** Results of Delong test for AUC comparisons among the training and two external validation cohorts.

|  | Delong test *P*-value |
| --- | --- |
| Training cohort vs External validation cohort 1 | 0.3969 |
| Training cohort vs External validation cohort 2 | 0.0631 |
| External validation cohort 1 vs External validation cohort 2 | 0.4422 |
